# Soil temperature, microbial biomass and enzyme activity are the critical factors affecting soil respiration in different soil layers in Ziwuling Mountains, China

**DOI:** 10.3389/fmicb.2023.1105723

**Published:** 2023-02-16

**Authors:** Ruosong Qu, Guanzhen Liu, Ming Yue, Gangsheng Wang, Changhui Peng, Kefeng Wang, Xiaoping Gao

**Affiliations:** ^1^Key Laboratory of Resource Biology and Biotechnology in Western China, Ministry of Education, Northwest University, Xi’an, China; ^2^College of Life Science, Northwest University, Xi’an, China; ^3^State Key Laboratory of Water Resources and Hydropower Engineering Sciences, Institute for Water-Carbon Cycles and Carbon Neutrality, Wuhan University, Wuhan, China; ^4^Department of Biology Sciences, Institute of Environment Sciences, University of Quebec at Montreal, Montreal, QC, Canada; ^5^Shuanglong State-Owned Ecological Experimental Forest Farm of Qiaoshan State-Owned Forestry Administration of Yan'an City, Yan'an, Shaanxi, China

**Keywords:** climate change, carbon cycle, soil microbial activity, microbial decomposition model, soil respiration (CO_2_)

## Abstract

Soil microorganisms are critical biological indicators for evaluating soil health and play a vital role in carbon (C)-climate feedback. In recent years, the accuracy of models in terms of predicting soil C pools has been improved by considering the involvement of microbes in the decomposition process in ecosystem models, but the parameter values of these models have been assumed by researchers without combining observed data with the models and without calibrating the microbial decomposition models. Here, we conducted an observational experiment from April 2021 to July 2022 in the Ziwuling Mountains, Loess Plateau, China, to explore the main influencing factors of soil respiration (R_S_) and determine which parameters can be incorporated into microbial decomposition models. The results showed that the R_S_ rate is significantly correlated with soil temperature (T_S_) and moisture (M_S_), indicating that T_S_ increases soil C loss. We attributed the non-significant correlation between R_S_ and soil microbial biomass carbon (MBC) to variations in microbial use efficiency, which mitigated ecosystem C loss by reducing the ability of microorganisms to decompose organic resources at high temperatures. The structural equation modeling (SEM) results demonstrated that T_S_, microbial biomass, and enzyme activity are crucial factors affecting soil microbial activity. Our study revealed the relations between T_S_, microbial biomass, enzyme activity, and R_S_, which had important scientific implications for constructing microbial decomposition models that predict soil microbial activity under climate change in the future. To better understand the relationship between soil dynamics and C emissions, it will be necessary to incorporate climate data as well as R_S_ and microbial parameters into microbial decomposition models, which will be important for soil conservation and reducing soil C loss in the Loess Plateau.

## 1. Introduction

The Intergovernmental Panel on Climate Change (IPCC) assessment reports that global average temperatures will rise by 2.1–3.5°C, and the frequency and intensity of extreme heatwaves and precipitation events are also likely to increase (Tollefson, 2021). This climate change is expected to put general stress on ecosystems. The soil ecosystem is an important part of the terrestrial ecosystem and the hub of material and energy flow in the biosphere (Piao et al., 2010b). Carbon (C) is the basic element of life forms, without which life cannot exist, so the C cycle is one of the most important biogeochemical cycles (Bot and Bernites, 2005), and terrestrial soil C cycle research is an important component of global change research. Soil microbes are largely involved in the soil C cycle and play a crucial role in climate feedback (Jansson and Hofmockel, 2020), including CO_2_, N_2_O, and other greenhouse gas emissions. As the most active component of soil, microorganisms are significant biological indicators for evaluating soil health (Fierer et al., 2021). In recent years, it has been proposed that soil microbial characteristics can be used as biological indicators of soil health to guide soil ecosystem management (Schloter et al., 2003; Cardoso et al., 2013). Sicardi et al. (2004) believe that soil microbial characteristics, such as soil respiration (R_S_), microbial biomass, and enzyme activity, vary significantly from season to season, suggesting that they are sensitive and reliable indicators of changes in soil physicochemical properties.

R_S_ refers to the process by which soil releases CO_2_ into the atmosphere and the most important component of R_S_ is the heterotrophic respiration of soil microorganisms (Wang et al., 2019). C is stored in the soil as organic matter, its storage is approximately twice that of the atmospheric C pool and it plays a significant role in the C cycle of the terrestrial ecosystem (Mahajan et al., 2021). Therefore, R_S_ can significantly affect the global C cycle in terrestrial ecosystems (Zhou et al., 2009). The world is now experiencing a period of rapid warming due to the effects of human activities and CO_2_ emissions, and R_S_, which releases more than 10 times more CO_2_ into the atmosphere than the combustion of fossil fuel (Marland, 1983), is the second-largest source of continental C fluxes (Hu et al., 2019). Due to the enormous storage capacity of soil organic carbon (SOC), even a small change in soil C storage and R_S_ will significantly affect the CO_2_ concentration in the atmosphere, thereby affecting the feedback effect of terrestrial ecosystems on climate change (Davidson et al., 2006).

In ecosystems, microorganisms play a crucial role in soil metabolism as decomposers that drive nutrient turnover in soil ecosystems by mineralizing organic matter (Wieder et al., 2015). Soil microbial biomass is the active component of soil organic matter (SOM) and the most active soil factor (Jenkinson and Ladd, 1981). Since soil microbial biomass is very sensitive to environmental factors, slight changes in soil can change it (Chander et al., 1998), so various environmental disturbances can be predicted earlier.

All soil biochemical processes proceed because soil enzymes act as the driving force. An essential soil microbial function is to decompose key nutrients in litter and accumulate organic matter through soil enzymes (Caldwell, 2005). For example, cellobiohydrolase (CBH) and β-1,4-glucosidase (βG) are required to decompose cellulose in a litter (Sinsabaugh et al., 1992), and peroxidase (PER) and polyphenol oxidase (PPO) also play important roles in lignin decomposition (Lucas et al., 2007). Green et al. demonstrated that oxidase is an important factor affecting soil microbial respiration (Green and Oleksyszyn, 2002). In addition, Sinsabaugh et al. (2008) also demonstrated that soil microbial biomass determines the organic matter decomposition process of soil enzymes. Therefore, soil enzyme activity and other soil microbial indicators can be used to identify early warnings of soil ecosystems under stress conditions and anthropogenic disturbances (Boerner R. et al., 2005).

The results of most ecosystem models show that climate change will stimulate the microbial decomposition of SOM and generate feedback on global climate change (Friedlingstein et al., 2006). The positive feedback system model for climate change over time has a poor effect in simulating the global SOC pool and has great uncertainty (Voigt et al., 2016). Therefore, the global ecosystem model needs to consider microbial effects to accurately predict the feedback relationship between climate warming and SOM decomposition (Ji et al., 2018). In recent researches, the accuracy of the models in predicting soil C pools has been improved by considering microbial involvement in the decomposition process in ecosystem models (Abs et al., 2020; Guo et al., 2020), but the parameter values of these models are assumed by researchers without integrating the observed data with the models and calibrating the microbial decomposition models. Therefore, to improve the accuracy of microbial ecosystem models, it is also necessary to calibrate microbial parameters, and R_S_, microbial biomass, and enzyme activity are the most reliable observations for model calibration and validation (Hanson et al., 2000; Wang et al., 2015). In addition, dynamic data (e.g., soil temperature and moisture) can represent real-world climatic and environmental conditions, which can be beneficial for the model and understanding soil C cycling more realistically (Wang et al., 2020).

Forest soil microorganisms, which are vital part of forest ecosystems, play an important role in the decomposition of litter and soil nutrient cycling (Barberan et al., 2015). Forest R_S_ occupies an important proportion of terrestrial ecosystems, and its dynamic changes will have an important impact on the global C balance (Laganière et al., 2012). Forest R_S_ is also one of the important research objects of the long-term monitoring CO_2_ flux network currently being established, which is of great significance to scientific ecology and earth system research (Schlesinger and Andrews, 2000).

The Loess Plateau is a mixture of arid, semiarid and semihumid areas but is generally considered a semiarid area (Yu et al., 2020) and has always been known for severe land degradation, low land productivity, and soil erosion (Fu et al., 2016). The Ziwuling Mountains are located in the hinterland of the Loess Plateau, which is a well-preserved natural secondary forest area that plays a critical role in improving the surrounding ecological environment and climate regulation (Kang et al., 2014). From April 2021 to July 2022, we carried out an observational experiment in the Ziwuling Mountains, Loess Plateau, China, to record the monthly diurnal changes in R_S_ and the monthly dynamic changes in soil microbial biomass and enzyme activity. Since soil physicochemical properties can vary significantly at different soil depths (Rahman et al., 2022), we collected topsoil (0–30 cm) and subsoil (30–100 cm) respectively in the process of collecting soil samples. We hypothesized that the topsoil and subsoil physicochemical and microbial properties would be significantly different, and soil microbial properties would also change significantly in different months or seasons. The main goals of this study were to I) explore the main influencing factors of R_S_ and II) determine which parameters can be incorporated into a microbial decomposition model.

## 2. Materials and methods

### 2.1. Study site

**Figure 1:**
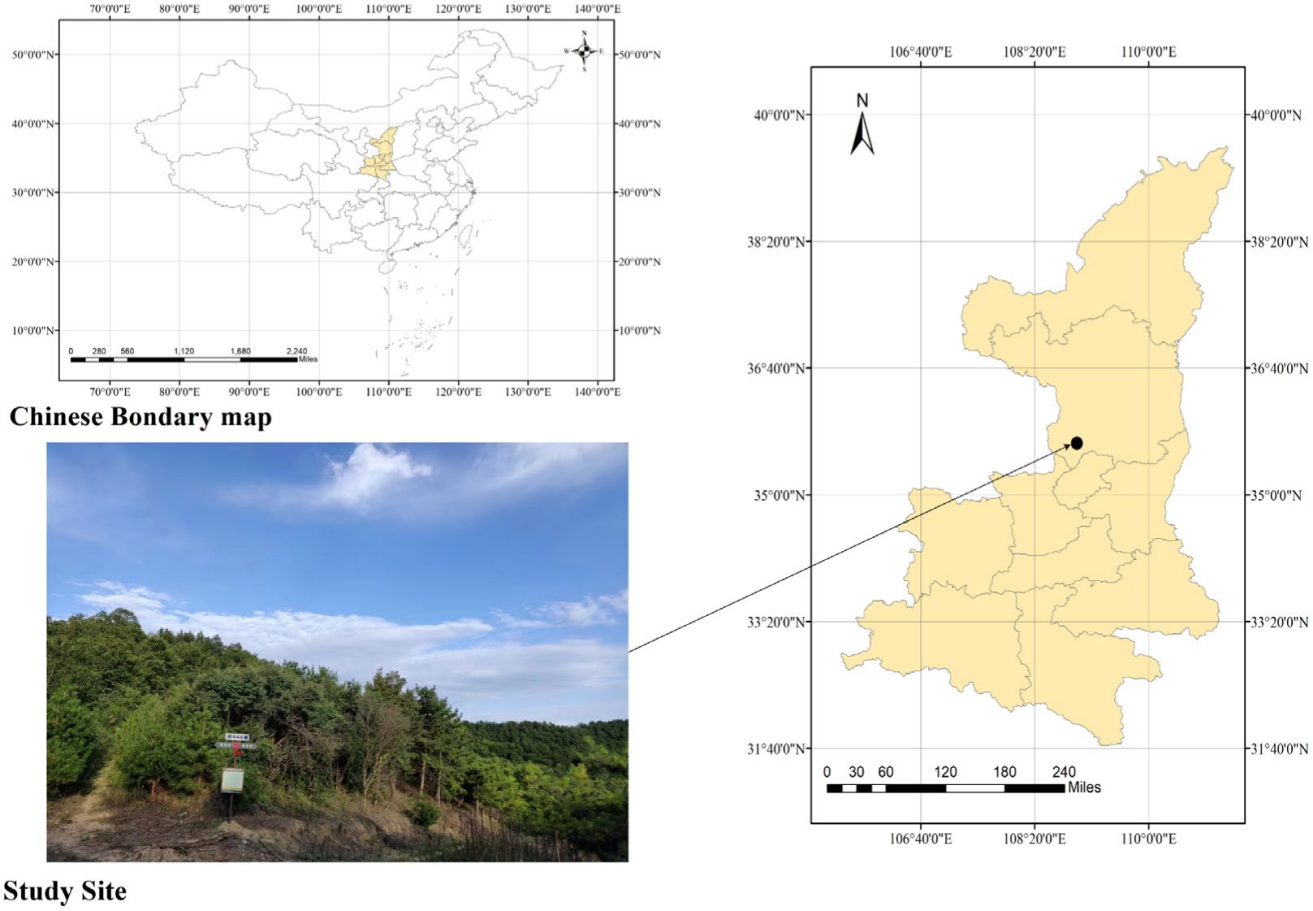
Map of study site. The Shuanglong Forest Farm is located in Shuanglong Town, Huangling County, Yan’an City, Shaanxi Province, China. It is located in the Ziwuling Mountains and is a natural secondary forest.

Field sampling and observation experiments were conducted from April 2021 to July 2022. The study site (Figure 1) was located in the Shuanglong Forest Farm (35°39′ ~ 35°43’N, 108°56′ ~ 108°58′E), a natural secondary forest in the Ziwuling Mountains of North China (Zhang et al., 2022). Our study site was 100 × 100 m. The climate of this site was a warm temperate semihumid climate, with a mean annual temperature of approximately 7.4°C and a mean annual precipitation of 587.6 mm (Chai et al., 2016). The main soil type was loessial soil, which was turbid brown or orange. The soil texture was loose and soft with few roots and pores, which indicated silt loam. The typical arbor species include *Betula platyphylla, Swida macrophylla, Carpinus turczaninowii Hance, Quercus aliena Bl, Quercus liaotungensis, Rhus potaninii Maxim, and cer davidii Franch*. The typical shrub species include *Acer tataricum subsp.ginnala, Viburnum dilatatum Thunb, Cotoneaster multiflorus Bge, Rhamnus leptophylla Schneid, Lonicera hispida Pall. ex Roem. et Schult*.

### 2.2. R_S_ observation experiment

Three sites with flat terrain were selected as sampling points for the measurement of the R_S_ rate (μ mol m^−2^ s^−1^). We installed ACE automatic R_S_ monitoring systems on iron rings with the inner diameter of 20 cm and the height of 10 cm (ACE-200, Ecotech Ecological Technology Ltd) and inserted 4–5 cm into the soil at each sampling point. R_S_ measurement sites were chosen to be more than 50 cm away from the surrounding vegetation, with each site being more than three meters away. To reduce soil disturbance, we inserted the iron rings at least 24 h before the measurement, and the broken roots and litter on the soil surface were removed. From April 2021 to July 2022, we used automatic R_S_ monitoring systems to monitor the R_S_ rate every 30 min for all sample points every month for 24 h. The R_S_ monitoring systems could simultaneously measure and record the soil temperature (T_S_, °C) and soil moisture (M_S_, %vol) within 0–10 cm below the surface soil of the sampling site. A meteorological monitoring station (CR200Series) was established at the research site to collect air temperature and moisture data from April 2021 to July 2022.

### 2.3. Soil sample collection

During the study period, from April 2021 to July 2022, soil sampling was performed every 2 months. Five sampling points were set at the research site using the five-point sampling method. To avoid edge effects, the sampling points were neither close to the edge of the plot nor far from the edge of the sample plot. Each sampling site was 5 × 0.5 m plot. The sampling sites were surrounded by abundant vegetation and the soil surface had obvious humus layers. We divided each sampling point into 12 areas, assigned a random block to all the sampling points, and conducted sampling according to the random block order (Supplementary Figure S1). The sampling depth was divided into two types. The soil at a depth of 0–30 cm below the surface soil was used as topsoil, and the soil at a depth of 30–100 cm was used as subsoil. A total of five replicates were collected separately for topsoil and subsoil. The soil from each depth at each sampling point was mixed evenly after collection, and the broken roots and litter in the soil were removed to reduce errors in the analysis process. Sterile gloves were worn during soil collection to prevent soil contamination. The soil samples were transported in sterile sampling bags, stored in a freezer, and taken to a laboratory by car for further analysis.

### 2.4. Soil sample analysis

We used the Kjeldahl method (Bradstreet, 1965) to determine the total soil nitrogen (TN), and the soil was hydrolyzed under alkaline conditions in a diffusion dish (Wang, 2010) to calculate the content of alkaline hydrolyzed nitrogen (HN). We used the alkali fusion-Mo-Sb antispectrophotometric method (Chen et al., 2018) to determine the total phosphorus (TP) and sodium bicarbonate solution (Cade-Menun and Lavkulich, 1997) to determine the available phosphorus (AP). The soil-available potassium (AK) was determined by ammonium acetate flame photometry (Zanati et al., 1973).The potassium dichromate oxidation-external heating method was used to determine soil organic matter (SOM), and then the SOM was determined by titration with a standard ferrous iron solution (Zhu et al., 2020). The soil pH was measured by using a pH meter. Microbial biomass carbon (MBC), microbial biomass nitrogen (MBN), and microbial biomass phosphorus (MBP) were determined by using the chloroform fumigation extraction method (Vance and Brookes, 1987). The soil samples were leached with KCL solution and then analyzed using a continuous flow analyzer to determine NH_4_^+^--N and NO_3_^−^--N (Liu et al., 2014).

We used a fluorometric method (Eivazi and Tabatabai, 1988) to measure the β-1,4-glucosidase (βG) activity in the soil and a nitrophenol colorimetric method (Wood and Bhat, 1988) to measure the cellobiohydrolase (CBH) activity. Polyphenol oxidase (PPO) was determined spectrophotometrically by using pyrogallol (1,2,3-trihydroxy benzene) as a substrate (Bach et al., 2013). Peroxidase (PER) was measured by calculating the rate of substrate oxidation after the addition of H_2_O_2_ (Burns et al., 2013).

### 2.5. Statistical analysis

The R_S_ mean value and error were calculated from three replicate measurements. The mean values and errors of soil physicochemical and microbial properties were calculated from five replicate measurements. Pearson correlation analysis was used to examine the correlation of R_S_ with T_S_ and M_S_. Origin 2017 software was used to obtain the regression equations between R_S_ rate, T_S_, and soil M_S_, and then these regression relationships were plotted. Monthly and seasonal differences in R_S_ and soil physicochemical and microbial properties were tested by ANOVA. The datasets were checked for normality and homogeneity assumptions before performing ANOVA. The magnitude of this feedback largely depends on the temperature sensitivity of SOM decomposition (Q_10_).

Q_10_ was measured by the exponential relationship between R_S_ and T_S_ and was calculated as follows:


(1) $$R_{S}={\mathrm{aebT}}_{S}$$

(2) $$Q_{10}=e^{10b}$$

where T_S_ is the soil temperature, a is the R_S_ rate when the soil temperature is 0°C, and b is the temperature coefficient reflecting the temperature sensitivity of R_S_.

To examine how soil microbial characteristics influenced R_S_, structural equation modeling (SEM) was performed with Amos software (IBM SPSS Amos 26.0.0) for different soil layers. In stepwise multiple regression (Supplementary Tables S1, S2), in order to optimize the model, we removed the non-significant variables and paths. We evaluated the goodness of fit of the model according to the low chi-square (χ^2^; the model is a great fit when 0 ≤ χ^2^/df ≤ 2) (Tabachnick and Fidell, 2007), the high whole-model *p* value (if *p* > 0.05, there is no path loss and the model was a great fit), the comparative fit index (CFI; the model is a great fit when 0.97 ≤ CFI ≤ 1) (Hu and Bentler, 1999), and a root mean square error of approximation (RMSEA; the model is a great fit when 0 ≤ RMSEA ≤0.05) (Vile et al., 2006).

## 3. Results

### 3.1. Atmospheric temperature and humidity observation values and soil physicochemical and microbial properties

**Figure 2:**
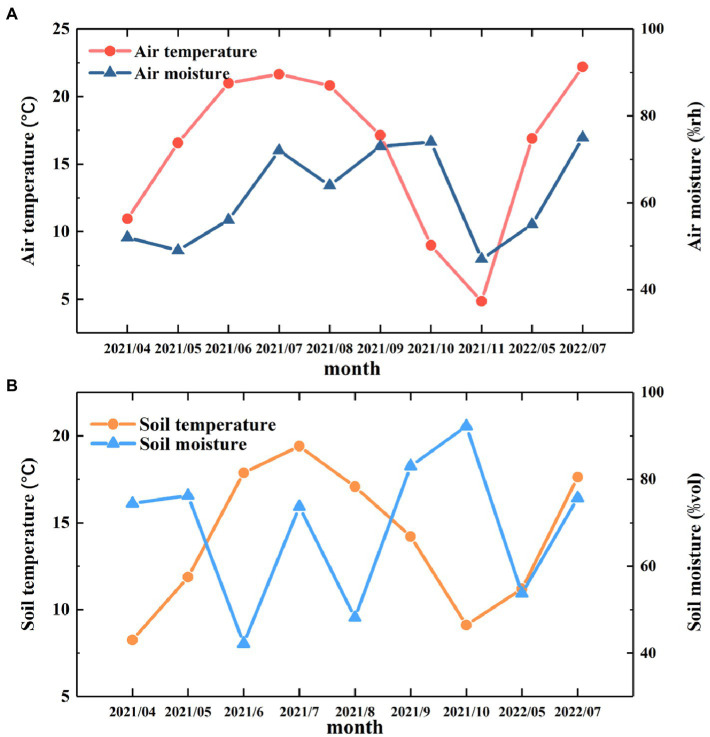
Monthly variations in temperature and moisture. **(A)** The monthly changes in air temperature and moisture from April 2021 to July 2022. **(B)** The monthly variations in soil temperature and moisture from April 2021 to July 2022.

The monthly variations in air temperature and air moisture are shown in Figure 2. During the observation period from April 2021 to July 2022, the average air temperature was 16.1°C, the highest temperature was 22.2°C, and the lowest temperature was 4.9°C. The average air moisture was 61.7%, the maximum moisture was 75.68%, and the minimum moisture was 46.70% (Figure 2A).

During the observation period, the T_S_ showed a pattern consistent with the seasonal variations in air temperature and air moisture. That is, the T_S_ gradually increased from April to July 2021, reaching a maximum value of 19.4°C in July 2021, and then the T_S_ decreased for the rest of the year. The M_S_ had obvious monthly variations during the observation period, reaching a maximum value in October 2021 and minimum value in June 2021 (Figure 2B). The maximum and minimum values were 92.44 and 42.20%, respectively.

The soil at the study site was alkaline, and there was no significant difference in pH between the topsoil (0–30 cm) and the subsoil (30–100 cm) (Table 1). The TP content in the subsoil was significantly higher than that in the topsoil, while the other soil physicochemical properties in the subsoil were lower than those in the topsoil, and AK and SOM were significantly reduced in the subsoil (*p* < 0.01).

**Table 1:** Soil physicochemical properties in the topsoil (0–30 cm) and subsoil (30–100 cm).

Variables	Soil layer	
	Topsoil	Subsoil
TN (g/kg)	1.91 ± 0.23	0.66 ± 0.03
TP (mg/kg)	385.06 ± 25.43**	474.61 ± 48.02**
HN (mg/kg)	194.91 ± 21.32	49.04 ± 2.73
AP (mg/kg)	6.27 ± 1.02	2.20 ± 0.49
AK (mg/kg)	208.84 ± 32.78**	107.67 ± 8.87**
SOM (g/kg)	27.77 ± 5.61**	16.99 ± 5.11**
pH	8.42 ± 0.02	8.69 ± 0.02

* denotes significant differences among different layers at *p* < 0.05. ** denotes significant differences among different layers at *p* < 0.01. TN, total nitrogen; TP, total phosphorus; HN, hydrolysable nitrogen; AP, available phosphorus; AK, available potassium; SOM, soil organic matter. The data are expressed as the mean values ± SEs.

### 3.2. Diurnal, monthly and seasonal variations in R_S_

The diurnal variation in the R_S_ rate in different months is shown in Supplementary Figure S2. The R_S_ rate showed a multi-peak distribution trend, and the R_S_ rate reached its peak at noon every day except in April and May within the 24 h observation period of each month. Except in June and August 2021, the R_S_ rate showed a minimum value in the morning (approximately 5:00 to 8:00 AM), and then the R_S_ rate gradually increased. The monthly variation in the R_S_ rate showed a trend of increasing and then decreasing. The R_S_ rate gradually increased after April, reached a maximum in July 2021, and then gradually decreased (Supplementary Figure S3).

**Figure 3:**
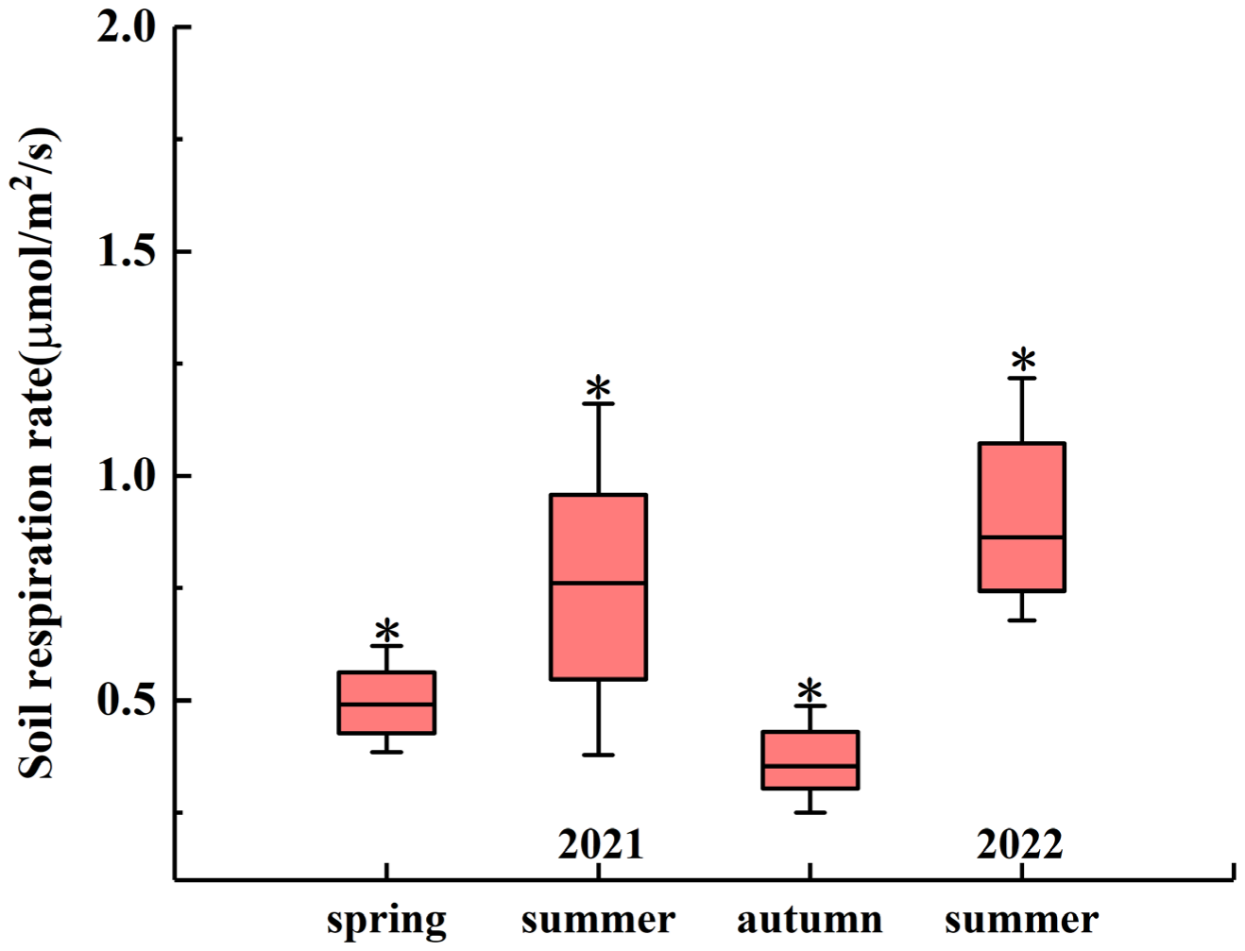
Seasonal R_S_ rates from April 2021 to July 2022. The asterisk (*) indicates significant differences in R_S_ rates between different seasons at a significance level of 0.05.

The seasonal changes in the R_S_ rate are shown in Figure 3. On the seasonal scale, there were significant differences between the R_S_ rates in different seasons. In 2021, the average R_S_ rate in summer was 0.75 μmol m^−2^ s^−1^, which was significantly higher than that in spring (0.52 μmol m^−2^ s^−1^) and autumn (0.37 μmol m^−2^ s^−1^) (*p* < 0.05). In 2022, the average R_S_ rate in summer was 0.97 μmol m^−2^ s^−1^ (Figure 3).

### 3.3. Relationship between T_S_, M_S_, and R_S_

**Figure 4:**
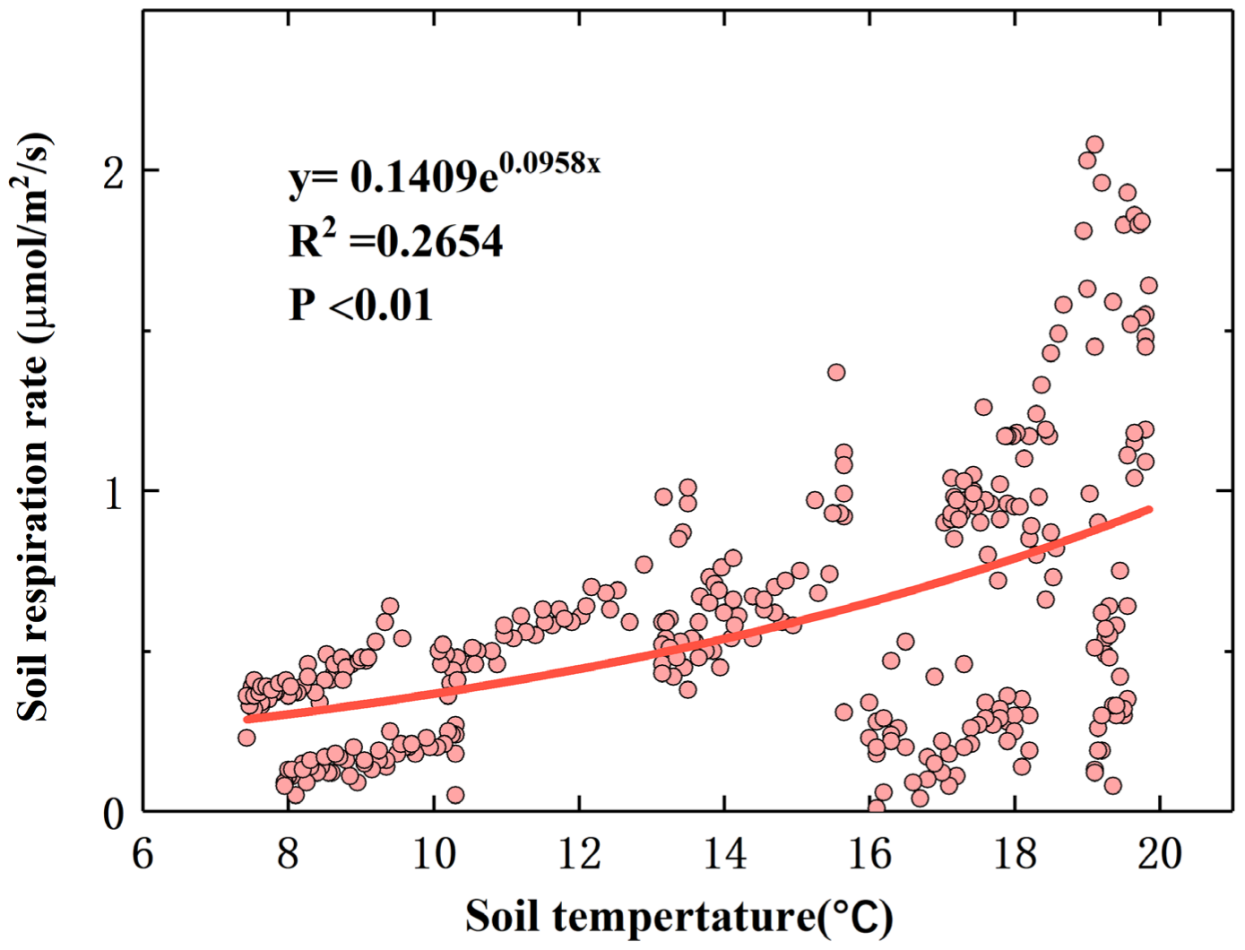
Relationship between R_S_ and soil temperature.

The correlation analysis results showed that there were significant correlations between T_S_, M_S_, and R_S_ (*p* < 0.01). The relationship between T_S_ and the linear equation fitting the diurnal-scale variation in R_S_ is shown in Figure 4, and the relationship between M_S_ and the linear equation fitting the diurnal-scale variation in R_S_ is shown in Supplementary Figure S4. The R_S_ rate increased with T_S_ and decreased with M_S_. According to Eq. (1), Q_10_ is 2.61, which is within the normal range (Zhou et al., 2009).

### 3.4. Soil inorganic nitrogen, microbial biomass, and enzyme activity

**Figure 5:**
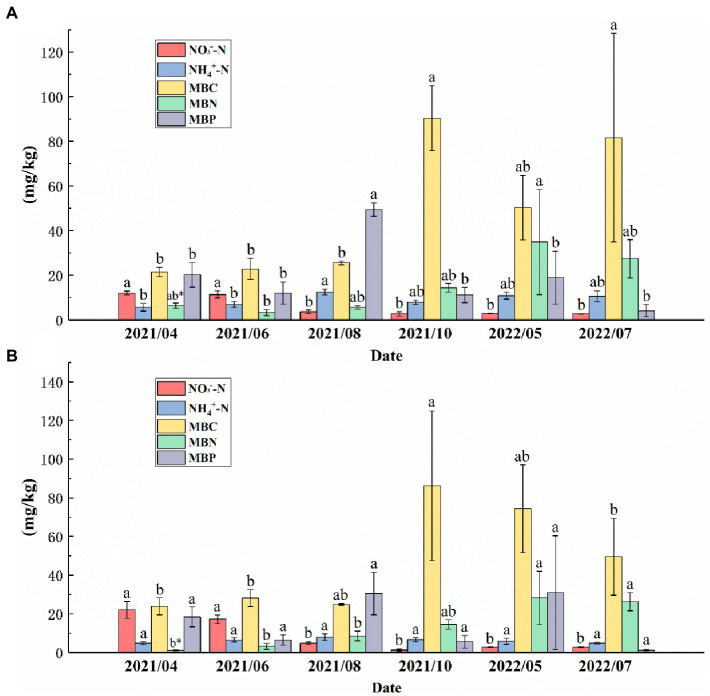
NH_4_^+^--N, NO_3_^−^--N, microbial biomass carbon (MBC), microbial biomass nitrogen (MBN), and microbial biomass phosphorus (MBP) contents in the **(A)** topsoil and **(B)** subsoil from April 2021 to July 2022. Different letters indicate significant differences between months for the same variable (*p* < 0.05).* indicates that the same variable in the same month is significantly different in different soil layers (*p* < 0.01).

In the topsoil, the NO_3_^−^--N from August 2021 to July 2022 was significantly higher than that from April to June 2021, and the MBC from October 2021 to July 2022 was significantly higher than that from April to August 2021. NH_4_^+^--N reached a maximum (12.59 mg/kg) in August 2021, and there were no significant differences in NH_4_^+^--N between other months except in August 2021. The MBP in the topsoil in August 2021 was significantly higher than that in the other months. The MBN reached the maximum (34.95 mg/kg) in May 2022 (Figure 5A). In the subsoil, the NO_3_^−^--N from April 2021 to June 2021 was significantly higher than that in other months, and there was no significant difference in NH_4_^+^--N among the 6 months. The MBC reached a maximum (113.88 mg/kg) in October 2021. The MBN from May to July 2022 was significantly higher than that from April 2021 to August 2021. There were no significant changes in MBP from April 2021 to July 2022 (Figure 5B).

The soil enzyme activity in the topsoil and subsoil varied significantly among different months (*p* < 0.05). The PER activity in the topsoil in May 2022 and July 2022 was significantly lower than that in the other months, and in the subsoil there were significant differences in the PER activity between April 2021 and July 2022. The PPO activity in both the topsoil and subsoil varied significantly among different months. Similar to that in the topsoil, in the subsoil, the βG activity in May 2022 was significantly higher than that in other months. The βG activity in the topsoil in April, June, and August 2021 was significantly different from the βG activity in the subsoil. The CBH activity in the topsoil in August 2021 was significantly lower than that in other months, and in the subsoil, the CBH activity in May 2022 was significantly higher than that from April to August 2021 but not significantly different from that in other months. In addition, from April 2021 to August 2021, the CBH and βG activities were significantly different in the topsoil and subsoil, so we believe that the CBH and βG activities in the topsoil were generally greater than those in the subsoil (Table 2).

**Table 2:** Peroxidase (PER), polyphenol oxidase (PPO), β-1,4-glucosidase (βG), and cellobiohydrolase (CBH) activities in the topsoil (0–30 cm) and subsoil (30–100 cm) from April 2021 to July 2022.

Site	Soil depth	PER (mg H_2_O_2_·g^−1^)	PPO (nmol·g-1·h^−1^)	βG (nmol·g-1·h^−1^)	CBH (nmol·g-1·h^−1^)
April-21	Topsoil	4.28 ± 0.05a	3687.34 ± 163.80a	181.65 ± 25.41b**	38.01 ± 13.33a**
June-21		3.97 ± 0.13a	3340.60 ± 76.59b	122.77 ± 14.06bc**	18.96 ± 4.66ab**
August-21		3.68 ± 0.40a	20.50 ± 1.20d	2.34 ± 0.35c**	0.18 ± 0.03c**
October-21		3.41 ± 0.29ab	476.30 ± 52.40c	73.92 ± 11.07c	16.01 ± 4.00bc
May-22		2.78 ± 0.37b	479.42 ± 52.89c	291.16 ± 42.54a	26.13 ± 2.42ab
July-22		2.28 ± 0.36b	496.76 ± 48.71c	199.42 ± 43.04b	28.75 ± 2.15ab*
April-21	Subsoil	4.10 ± 0.20a	2632.48 ± 219.10b	11.69 ± 2.86d**	9.79 ± 8.52b**
June-21		4.03 ± 0.25ab	3335.98 ± 156.34a	23.08 ± 9.38 cd**	1.62 ± 1.25b**
August-21		3.69 ± 0.12ab	16.84 ± 1.08d	0.31 ± 0.03d**	0.02 ± 0.01b**
October-21		3.21 ± 0.22ab	507.90 ± 49.44c	74.68 ± 11.48c	11.11 ± 4.84ab
May-22		3.23 ± 0.48ab	485.6 ± 41.14c	248.73 ± 20.69a	22.93 ± 2.45a
July-22		3.15 ± 0.50b	480.1 ± 63.26c	202.42 ± 39.42b	21.80 ± 0.49ab*

The data are expressed as the mean values ± SEs. Different letters indicate that the same variable differs significantly from month to month (*P* < 0.05).* indicates that the same variable in the same month is significantly different in different soil layers (*p* < 0.05). ** indicates that the same variable in the same month is significantly different in different soil layers (*p* < 0.01).

### 3.5. Relationship between soil microbial biomass, enzyme activity and R_S_

**Figure 6:**
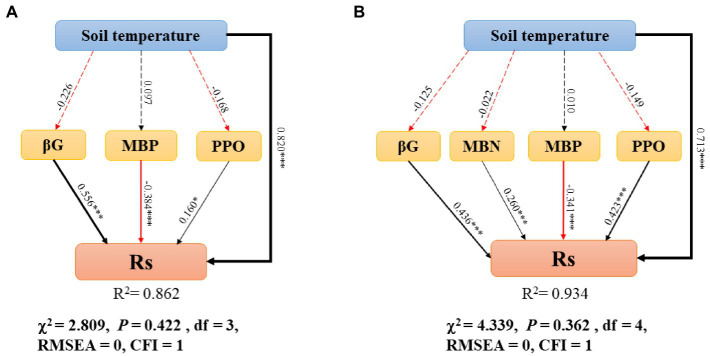
Structural equation model describes the relationship between variables and R_S_ in the topsoil **(A)** and whole soil **(B)**. R_S_, soil respiration; MBN, microbial biomass nitrogen; MBP, microbial biomass phosphorus; PPO, polyphenol oxidase; βG, β-1,4-glucosidase; soil temperature (T_S)_. Arrows represent the assumed direction of causation. The width of arrow is proportional to the path coefficient. The red and black solid lines represent negative and positive pathways, respectively. Insignificant pathways are indicated by grey dashed lines. The importance of the variables is reflected by standardized path coefficients. R^2^ reflects the proportion of variance explained for all variables in the model. The significance levels are as follows: *p < 0.05, **p < 0.01, and ***p < 0.001.

Based on the stepwise multiple regression results (Supplementary Tables S1, S2), we determined the variables that mostly explained the variation in R_S_. Model optimization was performed continually until the model fits well. SEM demonstrated the influence of R_S_ in different soil layers (Figure 6). The model for topsoil showed values of χ^2^ = 2.809, *p* = 0.422, df = 3, RMSEA = 0, and CFI = 1 (Figure 6A); the model for whole soil showed values of χ^2^ = 4.339, *p* = 0.362, df = 4, RMSEA = 0, and CFI = 1 (Figure 6B).

In the topsoil, T_S_, βG, MBP, and PPO all directly affected R_S_, except for MBP, T_S_, βG and PPO, which were significantly and positively correlated with R_S_, and T_S_, βG were the variables that had the strongest effects on R_S_. In the whole soil, T_S_, βG, MBP, MBN, and PPO all directly affected R_S_, except for MBP, T_S_, βG, PPO, MBN, which were significantly and positively correlated with R_S_, and T_S_, βG, PPO were the variables that had the strongest effects on R_S_.

## 4. Discussion

### 4.1. Effects of T_S_ and M_S_ on R_S_

Global warming not only increases the temperature of the atmosphere but also leads to changes in precipitation, which in turn causes greater variation in T_S_ and M_S_ (Zhang et al., 2016). In this study, the air temperature reached its highest value in summer and then gradually decreased, and the atmospheric moisture showed a multipeak trend, which experienced a decrease followed by an increase in the summer (Figure 1A). This may be related to the specific climate of the Loess Plateau. Since the Loess Plateau is an area sensitive to climate change, changes in atmospheric temperature and precipitation caused by global warming often lead to frequent droughts in many areas (Piao et al., 2010a). The climate of the Loess Plateau showed a trend of aridity in spring and summer (Hou et al., 2021) and then experienced violent precipitation in autumn, causing the air temperature to decrease after August, while air moisture began to increase significantly after August, reaching a maximum in October, and then gradually decreased again. T_S_ and M_S_ also show similar trends to air temperature and moisture (Figure 1B).

It has been demonstrated in previous studies that R_S_ is closely related to T_S_ (Wang et al., 2006). During the observation period, the monthly diurnal variation in R_S_ showed a multi-peak trend, and the R_S_ rate reached its peak value at noon and decreased to a minimum value in the morning (Supplementary Figure S2). This is consistent with the results of Wang et al. (2018). A possible explanation for this phenomenon is that the reaction process of R_S_ is mainly catalyzed by soil enzymes, and temperature is the main limiting factor affecting soil enzyme activity (Melillo et al., 2018). When the T_S_ is low, the activities of some critical enzymes that control R_S_ decrease, resulting in a lower R_S_ rate. On the monthly scale, with increasing T_S_, the R_S_ rate starts to increase in April, reaches a maximum value in July, and then gradually decreases to a lower value in October, which is consistent with the results of Tong et al. (2021). Our results differ from the results of Wen et al. (2018). A possible explanation for this phenomenon is the sensitivity of the research site to climate warming, resulting in a significantly higher rate of temperature change than in the other areas (Cao et al., 2016), thereby increasing the T_S_ to a maximum at an earlier time and leading to an increase in the R_S_ rate. The temperature sensitivity (Q_10_) of R_S_ is often used as an important parameter to measure the feedback of R_S_ to global warming (Hu et al., 2013). During the whole study period, there was a significant statistical relationship between the R_S_ rate and T_S_ (*p* < 0.01; Figure 4), and the fitting effect between the R_S_ rate and T_S_ also had some explanatory significance (*R*^2^ = 0.2654). Increased temperature stimulates R_S_ because climate warming may enhance the activity of soil microorganisms and promote soil organic C and litter decomposition, which partly explains why the R_S_ rate in summer is significantly higher than that in spring and autumn (Figure 3). Our findings suggest that a sustained increase in temperature may lead to greater soil C loss; that is, climate warming reduces soil C sinks.

M_S_ has been identified in previous studies as a major factor affecting R_S_, especially after drought rewetting events stimulate R_S_ (Hu et al., 2019). Our results show that M_S_ is significantly negatively correlated with R_S_ (Supplementary Figure S4), proving that M_S_ limits CO_2_ emissions from soil in a shorter period, which is different from the results of Yu et al. (2021). There may be several reasons for this: first, higher M_S_ inhibits the CO_2_ transportation process from the atmosphere to the soil (Yan et al., 2018). During the study period, the M_S_ variation range was 92.44% ~42.20%, especially in summer and autumn, and the M_S_ remained at a high level (Figure 2B). It has been suggested in previous studies that under anoxic conditions, soil organic carbon (SOC) may be more persistent (Li et al., 2021), resulting in a decrease in R_S_ rate. Second, differences in SOC and microbial communities that decompose specific soil SOC lead to different relationships between M_S_ and R_S_ (Yan et al., 2018). Third, in this study, M_S_ may not be the main factor affecting the R_S_ rate because R_S_ is often regulated by multiple factors (Duan et al., 2021). For example, soil salinity and R_S_ show a negative correlation. When in a salt-stress environment, the activities of plant roots and soil microorganisms are severely affected (Song et al., 2021).

The correlations between T_S_, M_S_, and R_S_ demonstrate that T_S_ and M_S_ data are useful for optimizing microbial decomposition models, which facilitates soil microbial activity prediction much better in the context of future climate change.

### 4.2. Physical and chemical properties in different soil layers

**Figure 7:**
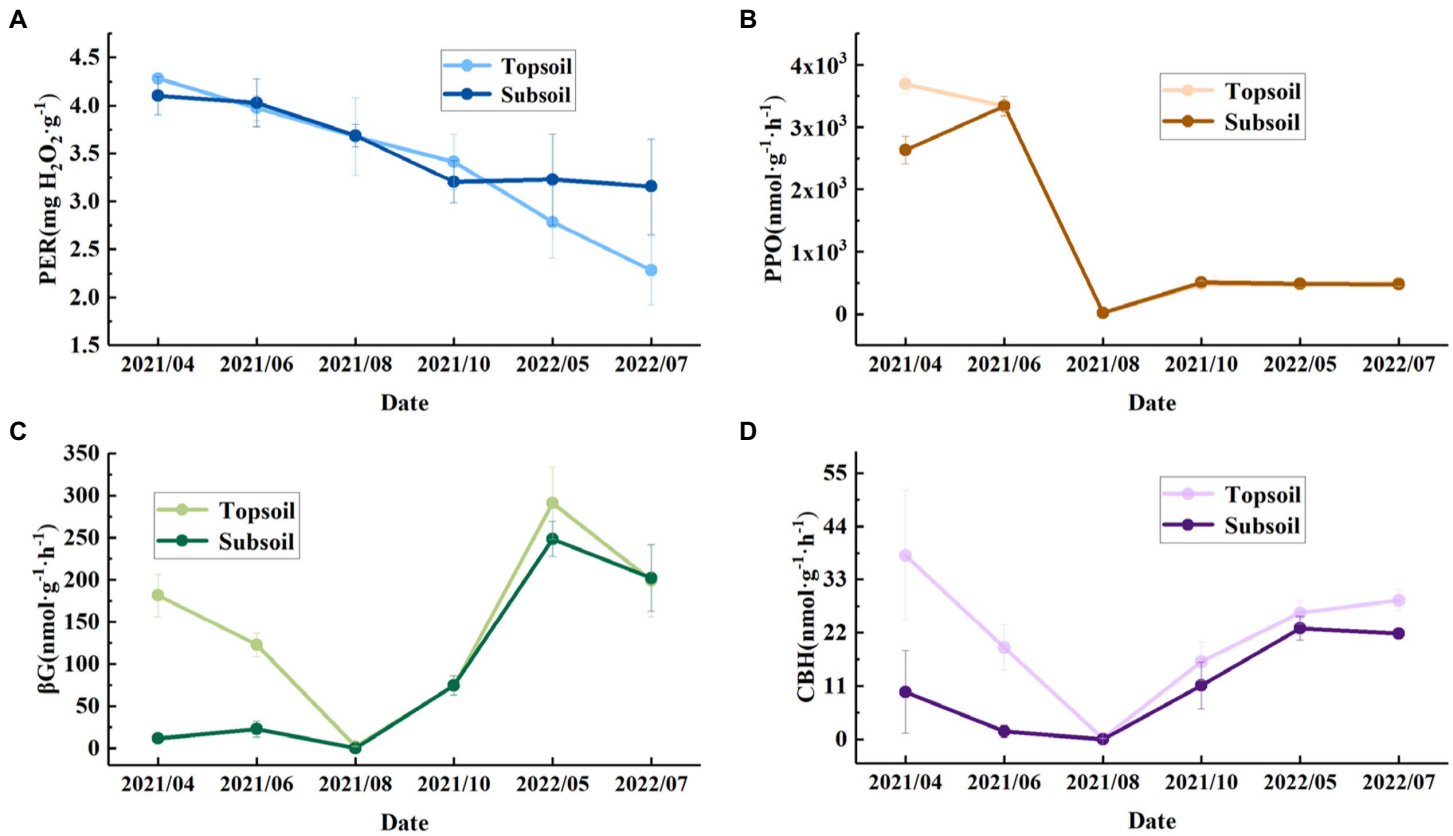
Monthly variations in enzyme activities in the topsoil (0–30 cm) and subsoil (30–100 cm) from April 2021 to July 2022. **(A)** PER, peroxidase. **(B)** PPO, polyphenol oxidase. **(C)** βG, β-1,4-glucosidase. **(D)** CBH, cellobiohydrolase.

Most previous studies have focused on the topsoil physicochemical properties (Liu et al., 2021). In this study, there were significant differences in the TP, AK, and SOM contents of different soil layers; TP was higher in the subsoil, and AK and SOM were higher in the topsoil (Table 1). The soil physicochemical properties changed with increasing soil depth, and our results are consistent with the results of Rahman et al. (2022). Microbial secretions can significantly affect soil potassium availability (Zorb et al., 2014), and factors such as microbial abundance and activity determine the pathway of soil litter conversion into SOM (Witzgall et al., 2021). In addition, plant roots and soil microbial communities can also dissolve P in the soil (Yang et al., 2021). Litter and most of the plant roots in the study site are concentrated in the topsoil. As shown in Figure 7, the microbial enzyme activity in the topsoil is higher than that in the subsoil, which proves that the microorganisms in the surface soil may be more active. Therefore, the AK and SOM contents were significantly higher than those in the subsoil, and the TP content was significantly lower than that in the subsoil. C and N cycle and nutrient turnover in soil are carried out by microorganisms through substrate (organic matter and litter) decomposition. In recent years, microbial decomposition models have been commonly used to explore the role of soil microorganisms in the coupled C and N cycle (Wang et al., 2014; Buchkowski et al., 2015). Our results can provide a reference for describing C, N, and P stocks and stoichiometry as well as soil nutrient distribution patterns in Loess Plateau soils and provide initial response data for soil microbial decomposition models.

### 4.3. Effects of soil microbial biomass and enzyme activity on R_S_

The soil microbial biomass can regulate microbial biochemical processes and nutrient cycling and affect soil physical and chemical properties, which in turn affects soil quality (He et al., 2003). MBC is often considered to be a crucial factor affecting R_S_, reflecting the ability of microorganisms to utilize SOC (Zeng et al., 2018). We found that, whether in the subsoil or topsoil, the MBC content showed a gradually increasing trend, and the MBC in May 2022 was significantly higher than in the other 3 months (Figure 5). We found that the trend of R_S_ was out of sync with that of the MBC. R_S_ decreased to a minimum value in autumn 2021, but the MBC reached a peak value in 2021. Our multiple stepwise regression results demonstrate that MBC is not a crucial factor affecting R_S_ (Supplementary Tables S1, S2). The reason for this phenomenon may be due to the decrease in the use efficiency of microorganisms. Microbial carbon use efficiency (CUE) refers to the distribution ratio between the MBC produced by organic matter catabolism and the C allocated by microorganisms for aerobic respiration (Schimel et al., 2022). The results of Manzoni et al. demonstrated that high temperature reduces the CUE of microorganisms (Manzoni et al., 2012) because the increase in T_S_ results in less C being allocated for microbial growth, which in turn reduces the ability of microbes to decompose organic resources to mitigate ecosystem C loss (Allison et al., 2010). During the growing season, T_S_ gradually increased, and microbes probably allocated more C for R_S_ than for MBC.

According to the results of the SEM, in addition to T_S_, βG and PPO also significantly influenced R_S_. Soil enzymes and microorganisms are involved in regulating the transformation of various organic matter and material circulation processes. Enzyme activity can be used as an indicator of microbial activity and plays a crucial role in decomposing organic compounds (Boerner R. E. J. et al., 2005). Previous studies have demonstrated that βG could participate in the decomposition of cellulose in litter (Caldwell, 2005). Litter is the most important source of organic matter input to the soil, and it can influence R_S_ by affecting the amount of labile C in the soil (Zhang et al., 2020). Therefore, βG has a significant positive effect on R_S_. In addition, PPO can promote the accumulation of SOC by depolymerizing or aggregating lignin molecules and phenolic compounds in the soil (KIRK et al., 1987), thus positively influencing R_S_.

Our results suggest that T_S_, microbial biomass, and enzyme activity may be the main factors influencing soil microbial activity, which has important scientific implications for constructing microbial decomposition models that predict soil microbial activity under climate change in the future. To better understand the relationship between soil dynamics and C emissions, it will be necessary to incorporate climate data as well as R_S_ and microbial parameters into microbial decomposition models, which will be important for soil conservation and reducing soil C loss in the Loess Plateau.

## 5. Conclusion

We examined the soil physical and chemical properties at different depths in the Ziwuling Mountains, Loess Plateau, China, and conducted a soil observation experiment to record the temporal and spatial dynamic changes in the soil microbial characteristics in this area. Our results prove that R_S_ in the Ziwuling area has obvious diurnal and seasonal variations and that the R_S_ rate is significantly correlated with T_S_ and M_S_. The strong effect of temperature on R_S_ leads to increased CO_2_ emissions from the soil to the atmosphere, which in turn leads to greater forest soil C loss. Our study reveals the main factors affecting R_S_, in order to better understand and predict changes in soil carbon dynamics in the future, incorporating T_S_, MBN, MBP, βG, and PPO data into microbial decomposition models is necessary.

## Data availability statement

The original contributions presented in the study are included in the article/Supplementary material, further inquiries can be directed to the corresponding author.

## Author contributions

KW conceived the idea and plotted the figures. RQ and GL conducted field sampling and sample determination. MY, GW, and CP conducted the statistical analysis. RQ and KW wrote the first draft of the manuscript. All authors contributed substantially to revisions, intellectual input and assistance to this study, and manuscript preparation.

## Funding

This research was supported in part by the National Natural Science Foundation of China (NSFC) (grant no. 41901059), Natural Science Foundation of Shaanxi Provincial Department (grant no. 2020JQ-593), and China Scholarship Council (CSC) (grant no: 202006970003).

## Conflict of interest

The authors declare that the research was conducted in the absence of any commercial or financial relationships that could be construed as a potential conflict of interest.

## Publisher’s note

All claims expressed in this article are solely those of the authors and do not necessarily represent those of their affiliated organizations, or those of the publisher, the editors and the reviewers. Any product that may be evaluated in this article, or claim that may be made by its manufacturer, is not guaranteed or endorsed by the publisher.
