# Supplementary material for: Soil temperature, microbial biomass and enzyme activity are the critical factors affecting soil respiration in different soil layers in Ziwuling Mountains, China
Source: Front Microbiol. 2023 Feb 16;14:1105723. doi: 10.3389/fmicb.2023.1105723 (PMC9978110; doi:10.3389/fmicb.2023.1105723)
Supplement: Supplementary file 1 [file Data_Sheet_1.docx]

Supplementary Material

**Soil temperature, microbial biomass and enzyme activity are the critical factors affecting soil respiration in different soil layers in Ziwuling Mountains, China**

Ruosong Qu^1^, Ming Yue^1^, Gangsheng Wang^2^, Changhui Peng^3^，Kefeng Wang^1*^

^1^College of Life Science, Northwest University, Xi’an, 710069, China

^2^Institute for Water-Carbon Cycles and Carbon Neutrality, and State Key Laboratory of Water Resources and Hydropower Engineering Sciences, Wuhan University, Wuhan 430072 China

^3^Department of Biology Sciences, Institute of Environment Sciences, University of Quebec at Montreal, C.P. 8888, Succ. Centre-Ville, Montreal H3C 3P8, Canada

* Correspondence: Corresponding Author: [wangkf@nwu.edu.cn](mailto:wanggs@whu.edu.cn)

# Supplementary Figures and Tables

**1.1 Supplementary Tables**

**Supplementary Table 1.** Results of stepwise multiple regression in topsoil. R_S_, soil respiration; MBP, microbial biomass phosphorus; PPO, polyphenol oxidase; βG, β-1,4-glucosidase; soil temperature (T_S_).

| Standardized coefficients | R^2^ | *F* | *P* |
| --- | --- | --- | --- |
| Rs= 0.591 T_S_ | 0.326 | 15.046 | 0.001 |
| Rs= 0.732 T_S_ + 0.623 βG | 0.697 | 34.412 | <0.001 |
| Rs= 0.748 T_S_ + 0.528 βG - 0.387 MBP | 0.841 | 52.216 | <0.001 |
| Rs= 0.769 T_S_ + 0.521 βG - 0.360 MBP + 0.150 PPO | 0.859 | 45.311 | <0.001 |

Dependent variable: R_S_

| **Supplementary Table 2.** Results of stepwise multiple regression in subsoil. R_S_, soil respiration; MBP, microbial biomass phosphorus; MBN, microbial biomass nitrogen; PPO, polyphenol oxidase; βG, β-1,4-glucosidase; soil temperature (T_S_).   \| Standardized coefficients \| R^2^ \| *F* \| *P* \| \| --- \| --- \| --- \| --- \| \| Rs= 0.579 T_S_ \| 0.310 \| 13.12 \| 0.001 \| \| Rs= 0.780 T_S_ + 0.622 CBH \| 0.657 \| 26.83 \| <0.001 \| \| Rs= 0.749 T_S_ + 0.515 CBH - 0.309 MBP \| 0.738 \| 26.37 \| <0.001 \| \| Rs= 0.749 T_S_ + 0.264 CBH - 0.320 MBP + 0.357 βG \| 0.809 \| 29.68 \| <0.001 \| \| Rs= 0.737 T_S_ + 0.152 CBH - 0.275 MBP + 0.490 βG + 0.299 PPO \| 0.892 \| 45.47 \| <0.001 \| \| Rs= 0.704 T_S_ - 0.299 MBP + 0.586 βG + 0.328 PPO \| 0.885 \| 52.82 \| <0.001 \| \| Rs= 0.704 T_S_ - 0.336 MBP + 0.431 βG + 0.417 PPO + 0.266 MBN \| 0.921 \| 64.27 \| <0.001 \| \| Dependent variable: R_S_ \| \| \| \| |
| --- | --- | --- | --- | --- | --- | --- | --- | --- | --- | --- | --- | --- | --- | --- | --- | --- | --- | --- | --- | --- | --- | --- | --- | --- | --- | --- | --- | --- | --- | --- | --- | --- | --- | --- | --- | --- |

## Supplementary Figures


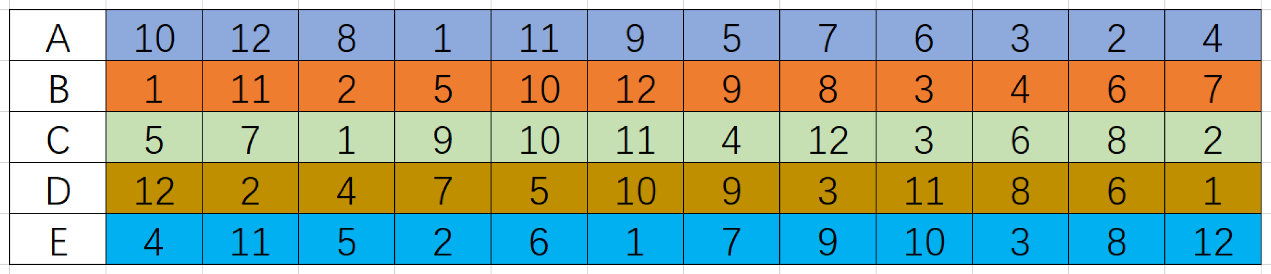


**Supplementary Figure 1.** Random block to all the sampling points.


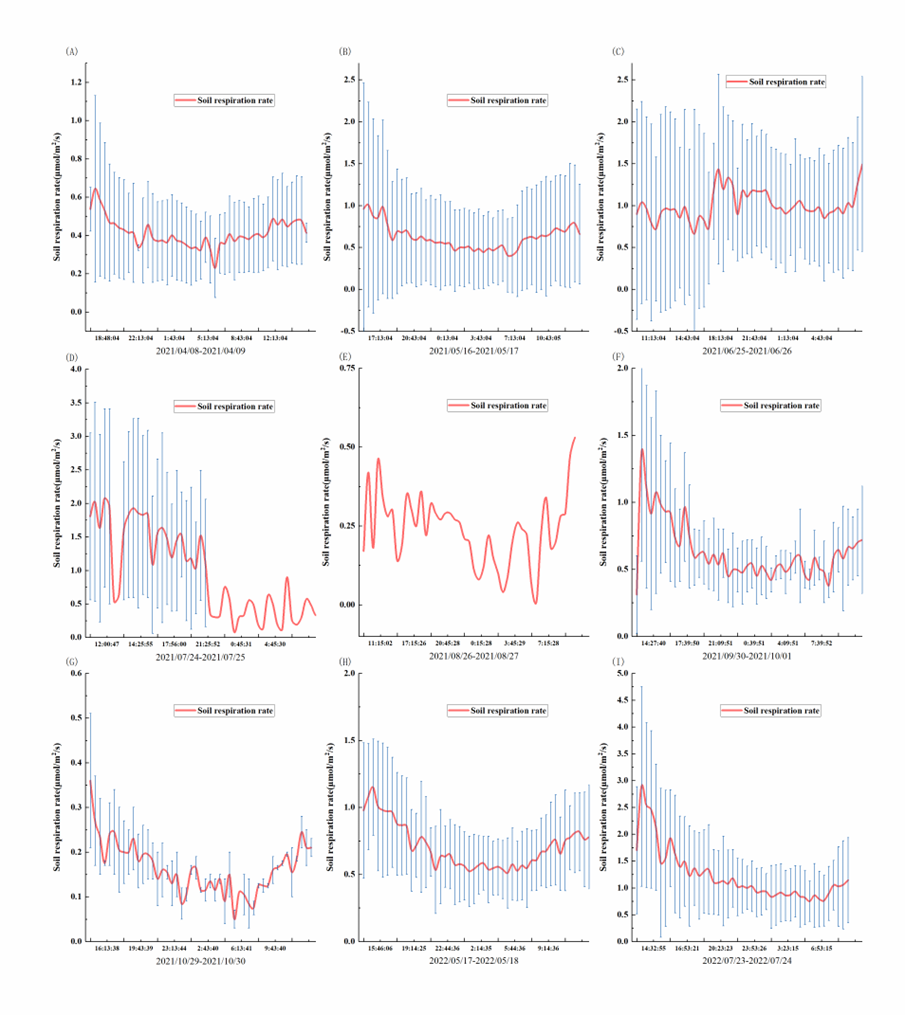
**Supplementary Figure 2.** Diurnal variation (A-I) of R_S_ rate (μmol m-2 s-1) from April 2021 to July 2022.





**Supplementary Figure 3.** Consecutive monthly changes of soil respiration from April to October 2021 and May to July 2022.





**Supplementary Figure 4.** Relationship between soil moisture and soil respiration during the observation period.
